# A Hand-Powered, Portable, Low-Cost Centrifuge for Diagnosing Anemia in Low-Resource Settings

**DOI:** 10.4269/ajtmh.2011.10-0399

**Published:** 2011-08-01

**Authors:** Jocelyn Brown, Lauren Theis, Lila Kerr, Nazima Zakhidova, Kelly O'Connor, Margaret Uthman, Z. Maria Oden, Rebecca Richards-Kortum

**Affiliations:** Rice University Institute for Global Health Technologies, Houston, Texas; University of Texas Health Science Center, Houston, Texas

## Abstract

This report describes the development of a hand-powered centrifuge to determine hematocrit values in low-resource settings. A hand-powered centrifuge was constructed by using a salad spinner. Hematocrit values were measured by using the hand-powered device, and results were compared with those of a benchtop centrifuge. The packed cell volume (PCV) measured with the hand-powered device correlated linearly with results obtained with a benchtop centrifuge (r = 0.986, *P* < 0.001). The PCVs measured with the hand-powered centrifuge were consistently 1.14 times higher than those measured with the benchtop system. The 14% increase in PCV measured with the hand-powered centrifuge is caused by increased plasma trapped in the cell column. The reader card was adjusted to compensate for trapped plasma. A hand-powered centrifuge and calibrated reader card can be constructed for U.S. $35 and can accurately determine hematocrit values. It is suitable for use in low-resource settings because it is mechanically-powered, inexpensive, and accurate.

## Introduction

Anemia poses a great threat to health in the developing world. Two billion persons, nearly one-third of the world's population, are anemic. Anemia accounts for significant morbidity and mortality in the developing world. Nutrient deficiencies and infectious diseases such as infection with human immunodeficiency virus, malaria, hookworm, and tuberculosis contribute to a high prevalence of anemia in poor areas.^1^ Currently, many cases of anemia remain undetected because health systems lack the funding, infrastructure, and training programs necessary to support basic diagnostic laboratory facilities to measure hematocrit or hemoglobin levels, particularly in rural areas.^2^

Methods to measure hemoglobin levels in low-resource settings include the World Health Organization color scale and the HemoCue Hemoglobin system (HemoCue AB, Ängelholm, Sweden). The World Health Organization color scale, although easy to use and affordable, is subject to error based on the reader's interpretation.^3^,^4^ The HemoCue system is more accurate, but relies on disposable cuvettes, is more expensive, and requires batteries, which are often unavailable or costly in low-resource settings.^3^,^4^ Benchtop centrifuges are the standard of care to measure hematocrit in industrialized nations, but like the HemoCue system, are often unavailable in low-resource settings because of power requirements and cost.

In developing regions, there remains an important unmet need for low-cost, portable tools that do not require electrical power to enable health care providers to accurately assess whether patients are anemic. This report presents a new technique to determine hematocrit that satisfies these criteria. We describe the development of a hand-powered centrifuge based on a commercial salad spinner. Results measured with the hand-powered centrifuge are consistent with those obtained by using a benchtop laboratory centrifuge across a wide range of hematocrit values.

## Materials and Methods

### Hand-powered centrifuge.

**Figure 1.**
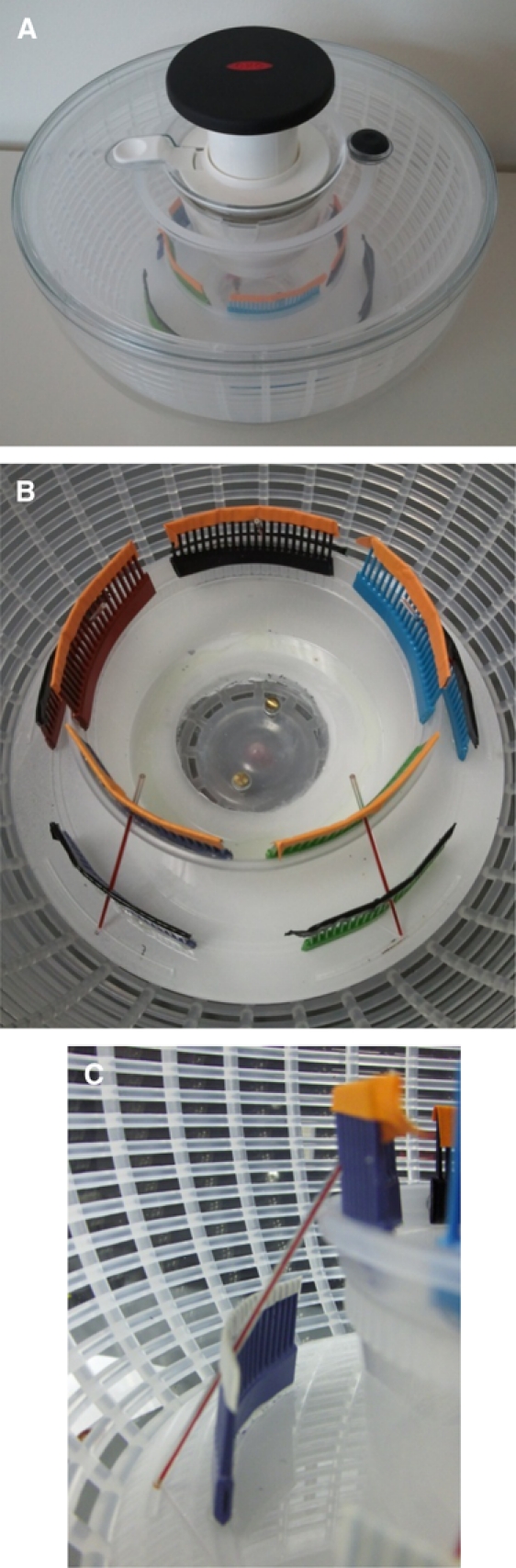
Hand-powered centrifuge: **A**, exterior; **B**, interior; and **C**, front view of microcapillary tube holder.

The hand-powered centrifuge design is based on a commercial salad spinner (OXO Good Grips Salad Spinner, retail price U.S. $29.99; OXO, Chambersburg, PA). With constant manual pumping, the basket rotated at an average of 600 revolutions per minute (RPM). An insert was designed to hold microcapillary tubes at an angle of 70° inside the salad spinner basket (Figure 1). A 17-cm diameter plastic circle was cut to serve as the base of the sample holder. A cylindrical plastic container with a diameter of 12 cm was glued to the center of the base. Five 6-cm segments of fine-toothed combs were glued to the plastic base and the inner lip of the cylindrical plastic container, forming two concentric circles. Tape was folded over the upper portion of each comb segment to ensure that the microcapillary tubes would be held securely in place. The insert was designed to hold microcapillary tubes between the comb teeth so that the angle between the plastic base and the microcapillary tubes was 70°. This angle does not need to be measured by the operator: when the top and bottom of the tube rests in the supports, the tube is held at a 70° angle. This angle is the same tube angle as a ZIPocrit Centrifuge (LW Scientific Inc., Lawrenceville, GA), which was used as the control for testing.^5^ Slots were etched into the plastic base to hold the bottom of the microcapillary tubes in place. When one tube is placed between every other pair of comb teeth, a maximum of 30 tubes can be accommodated simultaneously in the hand-powered centrifuge. The hand-powered centrifuge can be disinfected according to Biosafety Level 2 guidelines for laboratory-grade centrifuges, which specify that a spill in a centrifuge requires decontamination of the centrifuge and spill site by using an appropriate disinfectant, such as a 10% bleach solution.

### Testing the centrifuge.

#### Sample collection.

Venous blood samples (5 mL) were obtained from healthy men and women. Each participant gave written informed consent, and the study protocol was reviewed and approved by the Rice University Institutional Review Board (IRB). Blood was stored in a heparinized vial and used within three hours of collection. Blood samples (14 μL) were prepared in heparinized hematocrit capillary tubes (Chase Scientific Glass Inc., Rockwood, TN). The tubes were then sealed with Critoseal capillary tube sealant.

#### Pumping time determination.

Samples were obtained from one healthy woman according to the sample collection protocol described above. Two sealed microcapillary tubes were centrifuged for five minutes in a LW Scientific ZIPocrit centrifuge, as suggested in the LW Scientific ZIPocrit centrifuge user manual, to serve as reference values. Five additional sealed microcapillary tubes were loaded into the hand-powered centrifuge, one in each of the five comb segments so that the weight was balanced radially. To assess the time required for centrifugation, one operator pumped the hand-powered centrifuge for 24 minutes, and the packed cell volume (PCV) was determined for each sample at three-minute intervals. A metronome set at 120 beats per minute, which was determined to be the highest comfortable pumping rate for all operators, was used to provide an auditory guide to operators to maintain a consistent RPM. The hematocrit measurements were determined by measuring the PCV using the reader card that accompanied the ZIPocrit. The process was repeated for two additional operators; two samples were centrifuged in the ZIPocrit and five samples were centrifuged in the hand-powered centrifuge for each of the two additional operators.

#### User fatigue evaluation.

We evaluated user fatigue for 10 different users by using the 15-point Borg scale (ranging from no exertion at all to maximal exertion), which is commonly used to measure perceived exertion.^6^ Users reported their perceived level of fatigue at 0 minutes, 1 minute, 5 minutes, 9 minutes, and 10 minutes during the course of pumping the centrifuge.

#### Anemic sample simulation.

After determining the required centrifugation time, the performance of the hand-powered centrifuge was compared with that of the ZIPocrit by using blood samples with various hematocrit values. To simulate various degrees of anemia, whole blood from three participants was diluted with various amounts of compatible EDTA–plasma obtained from the Gulf Coast Regional Blood Center (Houston, TX). Five dilutions were prepared from each sample; fractions of plasma ranged from 20% to 100% in increments of 20%. Diluted samples were gently inverted for one minute to evenly mix the whole blood and plasma without lysing the erythrocytes. From each dilution, six 14-μL samples were prepared in microcapillary tubes as in the original procedure. One tube was centrifuged in the ZIPocrit for 5 minutes, and five tubes were centrifuged in the hand-powered centrifuge for 10 minutes. The process was repeated by a different operator nine times. Each operator was instructed to pump the hand-powered centrifuge at a constant RPM according to a metronome set at 120 beats per minute. Hematocrit values were determined for each sample by measuring the PCV for each sample using the ZIPocrit reader card.

#### Relative centrifugal force calculation.

The RPM of the hand-powered centrifuge was measured at 1 minute, 5 minutes, and 9 minutes within the 10-minute time span by using an Osprey-Talon Handheld Non-Contact Digital Laser/Photo Tachometer DT-2234A (Monarch Instrument, Amherst, NH). The RPM was converted to angular velocity, and the relative centrifugal force (RCF) in units of *g* exerted by the hand-powered centrifuge and the ZIPocrit were calculated according to the equation^7^ RCF = ω^2^r/980, where r is the radius in centimeters and ω is the angular velocity in radians/second.

#### Estimation of trapped plasma in the erythrocyte column.

We adapted the procedures of Chalin and Mollison^8^ and Bernstein^9^ to compare the fraction of plasma trapped in the erythrocyte column of samples centrifuged in the ZIPocrit and hand-powered centrifuges. Briefly, to 1 mL of fresh blood, we added 3 μL of Evans blue dye. Labeled blood was loaded into capillary tubes and centrifuged in either the ZIPocrit centrifuge for 5 minutes or in the hand-powered centrifuge for 10 minutes. After centrifuging, the tubes were cut to separate the plasma, buffy coat, and packed erythrocyte portions. The buffy coat fraction was discarded, and the plasma and packed erythrocyte portions were extracted and diluted 1:50 in phosphate-buffered saline. The optical density of each fraction was measured from 500 to 700 nm by using a Cary 5000 absorption spectrophotometer (Agilent Technologies, Palo Alto, CA). Each absorption spectrum was fit to a linear combination of hemoglobin, plasma, and Evans blue dye absorption by using a linear least squares fit to minimize the sum of the squared errors. The concentration of trapped plasma was calculated as the ratio of the Evans blue dye in the packed erythrocyte column to that in the plasma column and accounted for volume differences in the two column fractions. The process was repeated in triplicate and results were averaged.

#### Accuracy for patient specimens.

To assess the accuracy of hematocrit for patient specimens, whole blood samples were obtained from 47 persons. Venous blood was collected in heparinized tubes; anonymous specimens were obtained one week after collection. The protocol was reviewed and approved by the Rice University IRB and was found to be exempt from IRB review. From each vial, four 14-μL samples were prepared in microcapillary tubes. Two tubes were centrifuged in the ZIPocrit for 5 minutes, and two tubes were centrifuged in the hand-powered centrifuge for 10 minutes. The hand-powered centrifuge was filled with samples, and the operator pumped the centrifuge at a constant rate of 120 pumps per minute. Hematocrit values of the samples centrifuged in the ZIPocrit were determined for each sample by measuring the PCV for each sample using the ZIPocrit reader card, and hematocrit values of the samples centrifuged in the hand-powered centrifuge were determined for each sample by measuring the PCV for each sample using the reader card developed for the hand-powered centrifuge. Bland-Altman analysis was used to determine whether the difference between the two methods was related to the hematocrit value.

## Results

**Figure 2.**
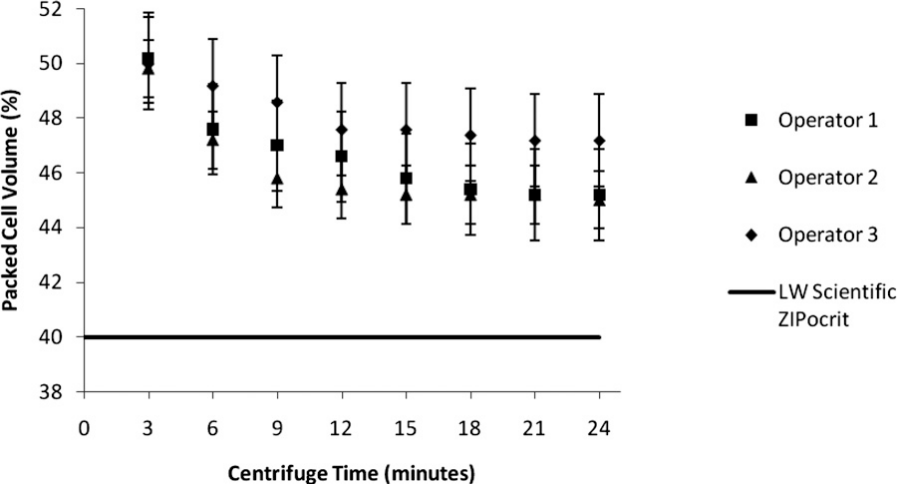
Measurement of packed cell volume (PCV) for whole blood from a woman for **A**, operator one; **B**, operator two; and **C**, operator three. Symbols show the average PCV measured for varying centrifugation times in the hand-powered centrifuge. Error bars show ±1 SD. The solid line indicates the reference PCV determined after five minutes in the ZIPocrit centrifuge.

Centrifugation of tubes containing whole blood in the hand-powered centrifuge resulted in successful separation of erythrocytes and plasma. To identify the required centrifugation time to fully pack erythrocytes in the hand-powered centrifuge, PCV was assessed at three-minute intervals over a 24-minute total centrifugation period. For all three users, the PCV decreased rapidly from 3 to 9 minutes (Figure 2). After 10 minutes of pumping, the PCV was within 21% of the PCV value determined by the ZIPocrit. Thus, all further experiments with the hand-powered centrifuge were conducted after a 10-minute centrifugation time. Because the PCV values determined by the ZIPocrit were the same value for each sample, no error bars are reported. On average, users rated fatigue experienced using the centrifuge as extremely light after 1 minute of pumping and very light after 10 minutes of pumping.

**Figure 3.**
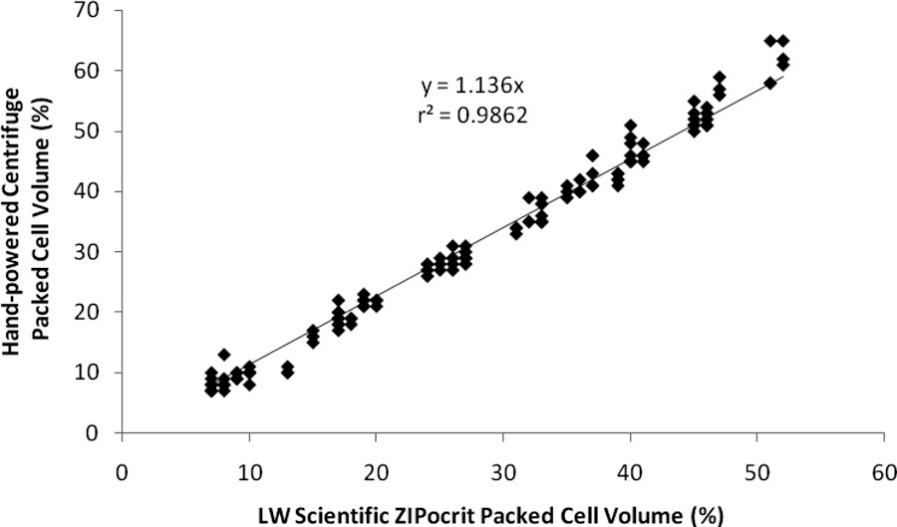
Measurement of packed cell volume (PCV) by using the hand-powered centrifuge with a 10-minute pumping time versus reference values determined by using the ZIPocrit centrifuge with a five-minute spin time. The PCV determined by using the hand-powered centrifuge is approximately 14% higher than that measured with the ZIPocrit for all tested degrees of anemia.

Performance of the hand-powered centrifuge was then compared with the ZIPocrit for samples prepared from whole blood samples diluted with various amounts of plasma. The PCV values for five operators who were women and operators who were men who used the hand-powered centrifuge were plotted against the reference values measured by using the ZIPocrit centrifuge and are shown in Figure 3. The PCV values obtained with the hand-powered centrifuge were consistently higher than those obtained with the ZIPocrit. Linear regression analysis showed a strong correlation between PCV values measured by using the two systems (r = 0.986, *P* < 0.001). The line of best fit for data from all operators (y = 1.136x) indicated that PCV values obtained with the hand-powered centrifuge were approximately 14% higher than those measured with the ZIPocrit across all hematocrit levels.

**Figure 4.**
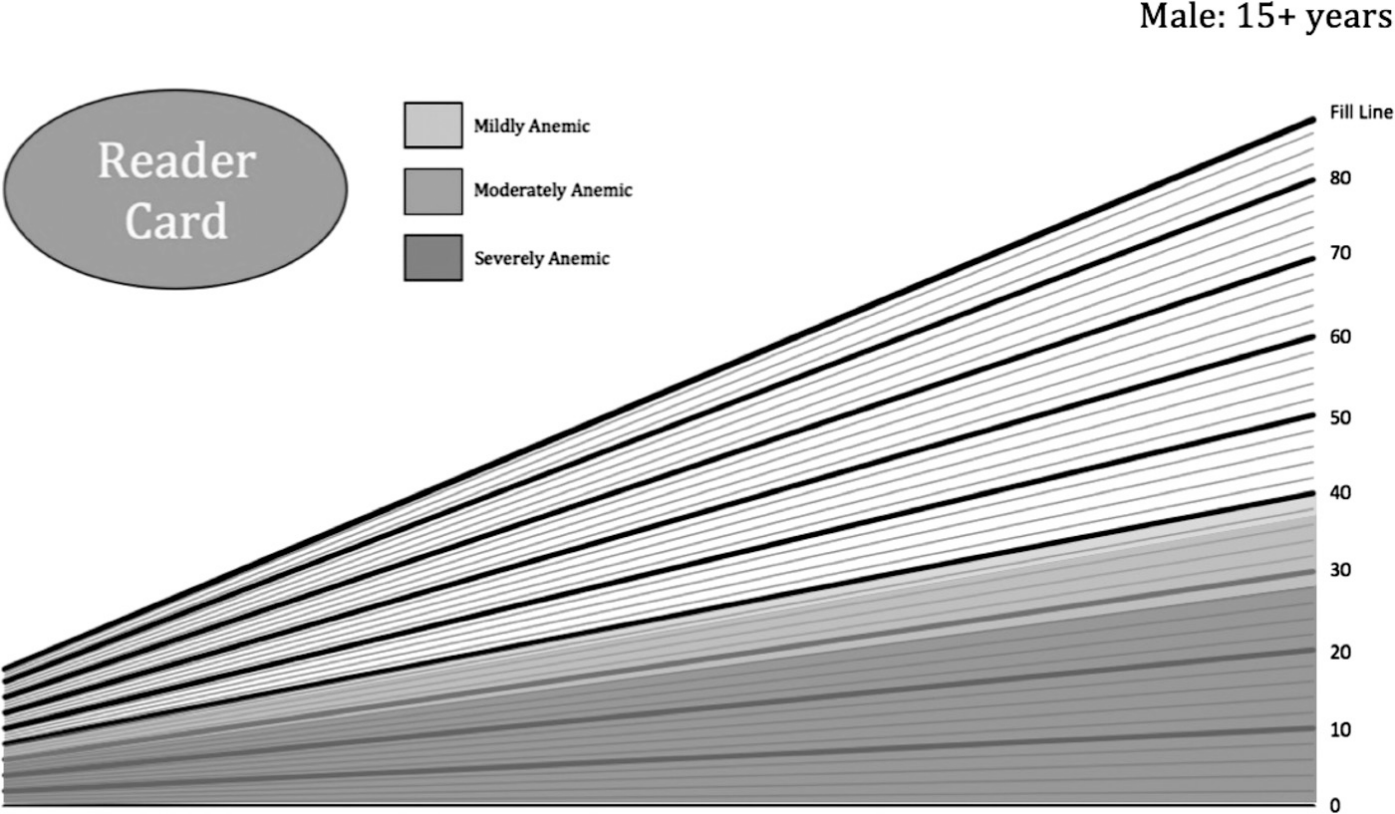
Adapted reader card for the hand-powered centrifuge. The slope of the lines converting packed cell volume to hematocrit are 1.14 times the slope of the lines on the standard reader cards on the basis of the calibration determined in Figure 3.

The reader card to convert between PCV and hematocrit values for the hand-powered centrifuge was adapted to account for the consistent differences in erythrocyte packing measured with the hand-powered centrifuge. The adapted reader card for the hand-powered centrifuge is shown in Figure 4. The slope of the lines indicating hematocrit values were adjusted to 1.14 times the slopes on the standard reader card on the basis of linear regression analysis described above. The adapted reader card was designed to easily accommodate standard microcapillary tubes, which have a height of 7.5 cm. It can be used for tubes up to 9.6 cm in height and for tubes filled to a minimum height of 2.9 cm. For tubes with an inside diameter of 0.5 mm, this corresponds to a minimum volume of 5.69 μL and a maximum volume of 18.85 μL. The card also denotes hematocrit regions that indicate the general anemia level of a patient.

To determine the boundaries of these classifications, the widely-accepted hemoglobin ranges were converted to hematocrit values by using the equation hematocrit (%) = (0.0485 × hemoglobin (mmol/L) + 0.0083) × 100.^10^,^11^ Because healthy hematocrit levels depend on age and sex of the person, several different reader cards were developed. When data from all users shown in Figure 3 were adjusted by using the reader card shown in Figure 4, the average absolute difference between measurements made with the hand-powered centrifuge and the ZIPocrit centrifuge was 1.76%.

The RPM of the hand-powered centrifuge was compared with the RPM of the ZIPocrit. The average RPM of the hand-powered centrifuge for nine users was 602 RPM (range = 400–730 RPM). The average RPM of the ZIPocrit was 11,000 RPM, as reported in the LW Scientific datasheet.^12^ The RCF of the hand-powered centrifuge was compared with the RCF of the ZIPocrit. The RCF of the hand-powered centrifuge was 31 *g*, and the RCF of the ZIPocrit was 4,370 *g*.

The erythrocyte fraction of samples prepared by using the ZIPocrit centrifuge contained a mean ± SD of 3.4% ± 1.4% trapped plasma, which is consistent with that of previous reports.^8^,^9^,^13^,^14^ The erythrocyte fraction of samples prepared by using the hand-powered centrifuge contained a mean ± SD of 17.6% ± 3.5% additional trapped plasma. This finding is consistent with the 1.146 ± 0.032 fold-increase in the PCV for these same samples prepared by using the hand-powered centrifuge relative to those prepared by using the ZIPocrit centrifuge.

**Figure 5.**
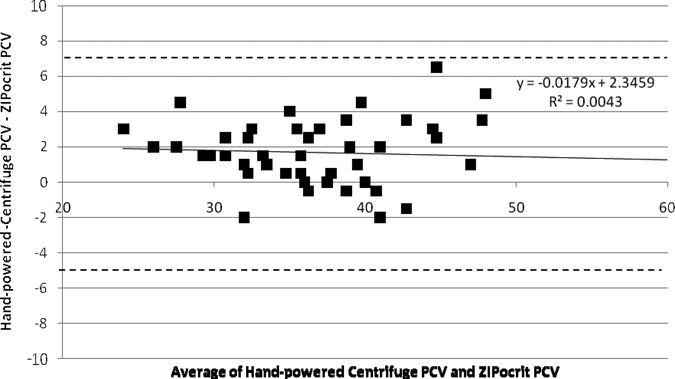
Difference between the packed cell volume (PCV) measured with the hand-powered centrifuge and that measured with the ZIPocrit versus the average of the PCV values measured with the hand-powered centrifuge and that measured with the ZIPocrit. Bland-Altman analysis of the data showed no linear correlation between the difference in PCV and average PCV for the two centrifuges. The mean difference was found to be approximately 2%, and the 95% limits of agreement are shown as dashed lines about the mean.

The PCVs from the 47 persons measured with the hand-powered centrifuge and the ZIPocrit were analyzed by using Bland Altman analysis. The difference between the PCV measured with the hand-powered centrifuge and that measured with the ZIPocrit versus the average of the two values for all 47 persons is shown in Figure 5. The mean difference was approximately 2%. The regression line of the difference in PCV measured with the two methods versus the mean showed no linear correlation, which indicated that the difference between the PCV measured with the hand-powered centrifuge and that measured with the ZIPocrit does not change with hematocrit.^15^

## Discussion

This paper reports a portable, low-cost, hand-powered centrifuge designed for use in low-resource settings. The centrifuge is inexpensive and easy to construct with a commercially available salad spinner. The materials for the device cost less than U.S. $35.00. The centrifuge weighs only 1.2 kg, and is small and light enough for transport between central health facilities and smaller villages. The device contains blood samples in microcapillary tubes and inside the salad spinner bowl. Should blood spill or a glass tube break, the biohazard and sharps risk are contained within the bowl, and the plastic surface of the salad spinner can be easily sanitized by using a 10% bleach solution, according to Biosafety Level 2 guidelines, without affecting the RPM of the hand-powered centrifuge.

The device is easy to operate. Users are instructed to depress the salad spinner pump at the same rate as the 120 beats per minute set on a metronome. On the basis of feedback from 10 operators, this pumping rate did not result in a significant increase in user fatigue over an elapsed time of 10 minutes. Each comb segment in the salad spinner can easily hold six microcapillary tubes, meaning that up to 30 samples can be accurately assessed at one time. When determining hematocrit for more than eight patients, the hand-powered centrifuge is more efficient than the ZIPocrit, which requires five minutes to centrifuge four microcapillary tubes.

Packing of erythrocytes was not as complete with the hand-powered centrifuge as with the ZIPocrit electrical bench top centrifuge. However, the ratio of PCV measured with the hand-powered centrifuge to that measured with the ZIPocrit was found to be constant across all hematocrit values tested. Thus, use of the hand-powered centrifuge and a calibrated reader card enabled accurate prediction of hematocrit values. Additionally, Bland-Altman analysis confirmed that the calibrated reader card enabled accurate prediction of hematocrit values across various hematocrit values. Differences in the PCV measured with the hand-powered centrifuge are likely caused by reduced centrifugal force generated in the hand-powered centrifuge.

Studies dating back to the 1950s have explored the relationship between centrifugal force, centrifuging time, and accuracy of hematocrit assessment.^8^,^9^,^12^,^13^ These studies showed that measuring the PCV leads to an overestimate of hematocrit because of trapping of small amounts of plasma between the erythrocytes in the packed erythrocyte column. The fraction of trapped plasma was higher for lower values of RCF and centrifugation time. To estimate the fraction of trapped plasma in the packed erythrocyte column, researchers have mixed blood samples with either small amounts of radioactively labeled protein^13^,^14^ or high molecular mass absorbing dye.^8^,^9^,^13^ The radioactivity or optical density of the packed cell column can be measured to assess the fraction of trapped plasma. Using samples labeled with Evans blue dye, Chalin and Mollison found that the fraction of plasma trapped in samples centrifuged at 1,500 × *g* decreased from 4.35% ± 0.62% at 30 minutes to 2.5% ± 0.13% at 55 minutes.^8^ Similarly, Bernstein found that the fraction of trapped plasma varied from 1.8% to 3.0% for samples centrifuged at 2,050 × *g* for 45–60 minutes.^9^ Using samples mixed with ^131^I-labeled albumin, England and others found that centrifuging samples at 12,000 × *g* for 5 minutes produced minimum plasma trapping that averaged 3%; no further reduction in trapped plasma was observed after this time.^14^ The fraction of trapped plasma found in samples prepared in the ZIPocrit centrifuge are consistent with these values. Moreover, the fraction of trapped plasma found in samples prepared with the hand-powered centrifuge accounts for the adjustment made to the reader card.

A similar device has been developed to separate serum from whole blood by using an egg beater.^16^ In this application, plastic tubing was used to hold blood samples instead of microcapillary tubes. The egg beater rotated the samples at approximately 1,200 RPM, which is twice the RPM achieved by the hand-powered centrifuge. However, despite the difference in RPM, we have determined that for the diagnosis of anemia, the hand-powered centrifuge achieves an appropriate rotational speed if proper adjustments are made to the reader card to account for trapped plasma.

Although the hand-powered centrifuge satisfies the constraints associated with use in the developing world and has proven effective in a controlled laboratory setting, its performance must still be assessed under clinical conditions by using a greater number of patient samples. Assessment of the useful lifetime of the hand-powered centrifuge, including durability, ability to maintain consistent rotational speed, user fatigue, and design scalability, must be considered. Limited human resources in low-resource settings could also pose a barrier to user adoption. On the basis of our testing, we believe the most fragile component of the hand-powered centrifuge is the insert that holds the samples. Future development of the insert, which would involve mass production of a custom insert that would function in the same way as the current prototype insert, will be required to improve its durability. With further field testing and possible modification, the hand-powered centrifuge has the potential to become an applicable tool to improve the diagnosis of anemia and related illnesses within the constraints of low-resource settings.
